# Risk of Acute Kidney Injury in Patients Randomized to a Restrictive Versus Liberal Approach to Red Blood Cell Transfusion in Cardiac Surgery: A Substudy Protocol of the Transfusion Requirements in Cardiac Surgery III Noninferiority Trial

**DOI:** 10.1177/2054358117749532

**Published:** 2018-01-03

**Authors:** Amit X. Garg, Nadine Shehata, Shay McGuinness, Richard Whitlock, Dean Fergusson, Ron Wald, Chirag Parikh, Sean M. Bagshaw, Boris Khanykin, Alex Gregory, Summer Syed, Gregory M. T. Hare, Meaghan S. Cuerden, Kevin E. Thorpe, Judith Hall, Subodh Verma, Pavel S. Roshanov, Jessica M. Sontrop, C. David. Mazer

**Affiliations:** 1 London Health Sciences Centre, Ontario, Canada; 2 Mount Sinai Hospital, University of Toronto, Ontario, Canada; 3 Cardiothoracic and Vascular Intensive Care Unit, Auckland, New Zealand; 4 Population Health Research Institute, Hamilton, Ontario, Canada; 5 Ottawa Hospital Research Institute, Ontario, Canada; 6 St. Michael’s Hospital, Toronto, Ontario, Canada; 7 Yale University, New Haven, Connecticut, USA; 8 Department of Critical Care Medicine, University of Alberta, Edmonton, Canada; 9 Rigshospitalet, Copenhagen University Hospital, Denmark; 10 Foothills Medical Centre, University of Calgary, Alberta, Canada; 11 McMaster University, Hamilton, Ontario, Canada; 12 St. Michael’s Hospital, University of Toronto, Ontario, Canada; 13 Dalla Lana School of Public Health, University of Toronto, Ontario, Canada; 14 Applied Health Research Centre, Li Ka Shing Knowledge Institute of St. Michael’s Hospital, Toronto, Ontario, Canada

**Keywords:** acute kidney injury, anemia, cardiac surgery, cardiopulmonary bypass, transfusion

## Abstract

**Background:**

When safe to do so, avoiding blood transfusions in cardiac surgery can avoid the risk of transfusion-related infections and other complications while protecting a scarce resource and reducing costs. This protocol describes a kidney substudy of the Transfusion Requirements in Cardiac Surgery III (TRICS-III) trial, a multinational noninferiority randomized controlled trial to determine whether the risk of major clinical outcomes in patients undergoing planned cardiac surgery with cardiopulmonary bypass is no greater with a restrictive versus liberal approach to red blood cell transfusion.

**Objective:**

The objective of this substudy is to determine whether the risk of acute kidney injury is no greater with a restrictive versus liberal approach to red blood cell transfusion, and whether this holds true in patients with and without preexisting chronic kidney disease.

**Design and Setting:**

Multinational noninferiority randomized controlled trial conducted in 73 centers in 19 countries (2014-2017).

**Patients:**

Patients (~4800) undergoing planned cardiac surgery with cardiopulmonary bypass.

**Measurements:**

The primary outcome of this substudy is perioperative acute kidney injury, defined as an acute rise in serum creatinine from the preoperative value (obtained in the 30-day period before surgery), where an acute rise is defined as ≥26.5 μmol/L in the first 48 hours after surgery or ≥50% in the first 7 days after surgery.

**Methods:**

We will report the absolute risk difference in acute kidney injury and the 95% confidence interval. We will repeat the primary analysis using alternative definitions of acute kidney injury, including staging definitions, and will examine effect modification by preexisting chronic kidney disease (defined as a preoperative estimated glomerular filtration rate [eGFR] <60 mL/min/1.73 m^2^).

**Limitations:**

It is not possible to blind patients or providers to the intervention; however, objective measures will be used to assess outcomes, and outcome assessors will be blinded to the intervention assignment.

**Results:**

Substudy results will be reported by the year 2018.

**Conclusions:**

This substudy will provide generalizable estimates of the risk of acute kidney injury of a restrictive versus liberal approach to red blood cell transfusion in the presence of anemia during cardiac surgery done with cardiopulmonary bypass.

**Trial Registration:**

www.clinicaltrials.gov; clinical trial registration number NCT 02042898.

## Abrégé

**Contexte:**

En chirurgie cardiaque, éviter les transfusions sanguines, lorsque sécuritaire, contribue à réduire les risques d’infections transfusionnelles et de complications tout en préservant une ressource rare et en réduisant le coût des soins. Ce protocole décrit une étude secondaire qui examine le volet des risques d’insuffisance rénale aiguë (IRA) de l’étude TRICS-III (Transfusion Requirements in Cardiac Surgery III), un essai de non-infériorité multinational, contrôlé et à répartition aléatoire faisant état des exigences applicables à la transfusion sanguine en chirurgie cardiaque. Notre protocole vise plus particulièrement à déterminer si le risque d’atteintes rénales encouru par les patients subissant une chirurgie de pontages coronarien est plus ou moins grand selon que l’on préconise une approche restrictive ou libérale en matière de transfusion sanguine.

**Objectif:**

L’objectif de notre étude est de déterminer si le risque d’IRA est plus ou moins grand selon que l’on préconise une approche restrictive ou libérale à l’égard de la transfusion sanguine; et, si c’est le cas, d’établir si ce risque différentiel perdure selon que le patient était ou non atteint d’insuffisance rénale chronique avant l’intervention.

**Cadre et type d’étude:**

L’étude consiste en un essai multinational de non-infériorité, contrôlé et à répartition aléatoire, mené entre 2014 et 2017 au sein de 73 centres répartis dans 19 pays.

**Patients:**

L’étude compte environ 4 800 patients ayant subi un pontage coronarien.

**Mesures:**

Le principal facteur observé est le développement d’une IRA périopératoire, telle que définie par une hausse marquée du taux de créatinine sérique par rapport à la valeur préopératoire (prélevée dans les 30 jours précédant l’intervention). Nous avons défini une « hausse marquée » par un taux de créatinine atteignant au moins 26,5μmol/L dans les 48 heures postopératoires ou son augmentation d’au moins 50 % à l’intérieur des sept premiers jours.

**Méthodologie:**

Nous présenterons le risque différentiel absolu d’IRA dans un intervalle de confiance à 95 %. Nous répéterons l’analyse primaire en alternant les définitions de l’IRA (notamment en fonction des différents stades) et examinerons les éventuelles modifications de l’incidence chez des patients atteints d’une insuffisance rénale préexistante (définie par un DFGe préopératoire à moins de 60 ml/min/1,73 m^2^).

**Limites de l’étude:**

Il n’est évidemment pas possible de procéder à l’insu des patients ni des fournisseurs de soins lors de l’intervention. Toutefois, des mesures objectives seront utilisées pour évaluer les résultats, et les évaluateurs ne seront aucunement au courant de la répartition des cas.

**Résultats:**

Les résultats de cette étude secondaire seront présentés d’ici 2018.

**Conclusion:**

Cette étude secondaire fournira des estimations généralisables du risque de développer une IRA lors d’un pontage coronarien selon que, pour traiter l’anémie, l’approche à l’égard de la transfusion sanguine soit restrictive ou libérale.

## What was known before

Initiating transfusions at a lower level of hemoglobin substantially reduces the number of transfusions performed with no adverse impact on clinical outcomes; however, uncertainty remains in the setting of cardiac surgery where patients may have less ability to tolerate anemia.

## What this adds

This substudy of Transfusion Requirements in Cardiac Surgery III (TRICS-III), a multinational noninferiority randomized controlled trial, will provide new knowledge on the risk of acute kidney injury of a restrictive versus liberal approach to red blood cell transfusion in the presence of anemia during cardiac surgery done with cardiopulmonary bypass.

## Introduction

Almost 20 million cardiac surgeries are performed worldwide each year, and the period during and shortly after cardiac surgery consumes 15% to 20% of the red blood cell supply available for transfusion.^1,2^ When safe to do so, avoiding blood transfusion in this setting protects a scarce resource, reduces costs, and avoids the risk of transfusion-related infection and other complications, which can include myocardial infarction, stroke, and acute kidney injury.^3,4^ However, the risks of transfusion must be balanced against the risks of untreated anemia, which can compromise tissue oxygenation and also increase the risk of myocardial infarction, stroke, and acute kidney injury.^5^

Avoiding unnecessary red blood cell transfusions in cardiac surgery has been identified as a critical area for research.^2^ Emerging evidence suggests that initiating transfusions at a lower level of hemoglobin substantially reduces the number of transfusions performed with no adverse impact on clinical outcomes.^4,6^ These data come from randomized controlled trials as it is impossible to reliably disentangle the adverse effects of anemia and red blood cell transfusion in observational studies. In controlled trials, patients were randomized to receive a restrictive versus liberal approach to transfusion initiation, where initiation occurred at a lower versus higher level of hemoglobin, typically 75 g/L versus 95 g/L, respectively.^4,6^ Meta-analyses of trials examining these approaches have been done in the nonsurgical and surgical setting, including small trials of patients undergoing cardiac surgery. While these studies suggest that a restrictive versus liberal approach to red blood cell transfusion is safe,^4,6-9^ uncertainty remains in the setting of cardiac surgery where patients may have less ability to tolerate anemia, and where trials conducted to date have been too small to determine whether there are meaningful differences in outcomes between the 2 groups.^4,6,8,10^

Prompted by the need for better evidence, the Canadian, Australian, and New Zealand governments provided funding for a noninferiority multicenter randomized controlled trial to test the hypothesis that the risk of adverse clinical outcomes from cardiac surgery with cardiopulmonary bypass is no greater with a restrictive versus liberal approach to red blood cell transfusion. The Transfusion Requirements in Cardiac Surgery III (TRICS-III) trial (NCT02042898) was launched in 2013 and includes over 4800 patients. The primary outcome of adverse events from cardiac surgery is a composite of all-cause mortality, myocardial infarction, new focal neurological deficit, and new kidney failure treated with dialysis, as assessed within 28 days of surgery or at hospital discharge, whichever comes first.

Approximately 1% of patients who undergo cardiac surgery will develop severe acute kidney injury and receive dialysis in the perioperative period. Many more patients (over 15%) will develop an important degree of acute kidney injury^11-13^ that is not treated with dialysis but is still associated with short- and long-term mortality, a longer hospital stay, and higher health care costs.^11,14-17^ This evidence prompted us to develop a kidney substudy of the TRICS-III trial. Our substudy will test the hypothesis that the risk of perioperative acute kidney injury is no greater with a restrictive approach to red blood cell transfusion during cardiac surgery than a liberal approach. We also want to know whether this holds true in patients with and without preexisting chronic kidney disease. This protocol describes our plan to conduct these kidney substudy analyses in TRICS-III.

### Primary Question

1. Among patients undergoing planned cardiac surgery with cardiopulmonary bypass, is a restrictive approach to red blood cell transfusion noninferior to a liberal approach with respect to the risk of perioperative acute kidney injury?

### Secondary Question

2. Does the presence of preexisting chronic kidney disease modify the effect of a restrictive versus liberal approach to transfusion with respect to the risk of acute kidney injury?

## Methods

### The TRICS-III Trial

TRICS-III (NCT02042898) is a multinational, open-label randomized controlled trial testing 2 commonly used red blood cell transfusion approaches in high-risk patients undergoing planned cardiac surgery with cardiopulmonary bypass (recruitment period 2014 to 2017; funded by the Canadian Institutes of Health Research, the National Health and Medical Research Council of Australia, and the Health Research Council of New Zealand). The prespecified methods of this large international trial are described elsewhere (available from the authors upon request).^18^ Briefly, this parallel group, pragmatic, noninferiority trial was designed to evaluate whether a restrictive approach to transfusion is no worse than a liberal approach with respect to a composite primary outcome of all-cause mortality, myocardial infarction, new focal neurological deficit, and new kidney failure treated with dialysis. The primary outcome will be assessed up to postoperative day 28 or at hospital discharge, whichever comes first. Planned enrollment was 5000 patients, and the trial accrual ended in March 2017 with 5028 randomized patients across 73 centers in 19 countries (as described below, 4850 randomized participants completed surgery). The data are currently being prepared for analysis (no between-group outcome analyses have yet taken place).

#### Randomization and intervention

Patients were identified in the preoperative anesthesia or cardiac surgery clinic or in the in-patient unit at each hospital, where informed consent was obtained. Enrolled patients were randomized to receive a restrictive or liberal approach to red blood cell transfusion. Randomization was generated centrally online by the Applied Health Research Centre at the Li Ka Shing Knowledge Institute of St. Michael’s Hospital (Toronto, Ontario, Canada). Randomization was stratified by center using random permuted blocks of varying sizes. For the restrictive approach, transfusion was not initiated until a patient’s hemoglobin concentration fell below 75 g/L intraoperatively and/or postoperatively in the intensive care unit or on the hospital ward. For the liberal approach, transfusion was initiated once a patient’s hemoglobin concentration fell below 95 g/L intraoperatively or postoperatively in the intensive care unit or if it fell below 85 g/L on the ward. For each group, transfusion occurred within the following time frames of the hemoglobin trigger: within 2 hours for patients in the operating room, within 18 hours for patients in the intensive care unit, and within 40 hours for patients on the hospital ward.

### Methods Used in the TRICS-III Kidney Substudy

Ethics approval was obtained for additional renal data collection at all 73 participating centers (in 19 countries). Key aspects of the TRICS-III protocol that are relevant to the proposed kidney substudy are described below.

### Patient Selection

Inclusion criteria for the TRICS-III main trial were as follows: adults aged 18 years or older undergoing a planned cardiac surgery with cardiopulmonary bypass, and a Preoperative European System for Cardiac Operative Risk Evaluation (EuroSCORE I) of 6 or more. Exclusion criteria were patient refusal or inability to receive blood products, involvement in the autologous predonation program, having a heart transplant, having a type of surgery solely for an insertion of a ventricular assist device, and the presence of pregnancy or lactation. Patients who were randomized but who did not undergo cardiac surgery will be excluded from the analysis (we expect ~3% of randomized patients did not undergo surgery).

The following additional exclusions will be applied to the TRICS-III kidney substudy:

1. Patients with preoperative end-stage kidney disease, defined as previous chronic dialysis, receipt of a kidney transplant, or an estimated glomerular filtration rate (eGFR) <15 mL/min per 1.73 m^2^ (these patients will be excluded because the prevention of postoperative acute kidney injury is no longer relevant; we expect <2% of randomized patients to be excluded for this reason^19^).
2. Patients missing a preoperative serum creatinine value (because preoperative serum creatinine is necessary to identify acute kidney injury, which is defined by an acute increase in serum creatinine from the preoperative value; we expect <1% of randomized patients will be missing a preoperative serum creatinine value).
3. Patients having *emergency* cardiac surgery will be excluded for 2 reasons: (1) They frequently have acute kidney injury or other complications prior to surgery, and so the preoperative serum creatinine may not be stable at baseline and (2) unfortunately many die, either in the operating room or shortly thereafter, before postoperative acute kidney injury develops (we expect <1% of randomized patients will have undergone emergency cardiac surgery).

### Data Collection and Outcomes

Preoperative serum creatinine was obtained within the 30-day period before surgery, where the most recent value before surgery will serve as the baseline value. Participating sites received study funds to measure the postoperative serum creatinine on days 1, 2, 3, and 5 after surgery. To our knowledge, at participating sites, serum creatinine measurements were traceable to isotope dilution mass spectrometry. No urine output data will be used in the primary analysis of this substudy given difficulties with accurately measuring this variable outside of the intensive care unit.

The primary outcome of the TRICS-III kidney substudy is perioperative acute kidney injury, defined as an acute rise in serum creatinine from the preoperative baseline value, where an acute rise is defined as ≥26.5 μmol/L in the first 48 hours after surgery or ≥50% in the first 7 days after surgery.^20^ Results from an earlier cardiac study with similar enrollment criteria as TRICS-III suggests that our data collection schedule will capture most perioperative acute kidney injury events in the first 5 days of surgery; in this previously published prospective surgery study of 1219 cardiac patients, 15% of patients developed perioperative acute kidney injury (as per our definition); 13.9% of events were observed in the hours following surgery and 38.7%, 38.7%, and 8.7% of events were observed on postoperative days 1, 2 and 3, respectively.^21^ For this reason, our protocol provides each center funding for serum creatinine measures on days 1, 2, 3, and 5 to encourage measurement on these days in all patients; as well, any other serum creatinine measures obtained in the perioperative period as part of routine care are recorded in the trial database (recording both their value and their date). We will compare the number of serum creatinine measurements (and the postoperative days of these measurements) in each intervention group during the hospital stay to examine the potential for ascertainment bias. While health care providers in TRICS-III are aware of the intervention received, this was also the case in our analyses of acute kidney injury within a large randomized trial where coronary artery bypass surgery was done with and without a bypass pump (where there was no difference in the frequency of serum creatinine measurement between the 2 groups given how ubiquitous serum creatinine measurements are in routine care in the perioperative setting^19^). We expect that less than 2% of patients will be missing serum creatinine values in the first 2 days of surgery as a result of death.

### Statistical Power

The TRICS-III study is designed to test whether a restrictive approach to transfusion is noninferior to a liberal approach. The total required sample size for the TRICS-III kidney substudy is 3378 based on a noninferiority margin of 3.5% for an absolute risk increase in acute kidney injury with the restrictive versus liberal approach, a 1-sided alpha of 0.025, and 85% power, assuming a baseline rate of acute kidney injury of 15%, <2% missing data due to death, and ~85% meeting our eligibility criteria. Thus, our projected available sample size of 4074 patients for this substudy will be adequate. A noninferiority margin of 3.5% is a reasonable minimal clinically important difference; for example, in our prior analyses of acute kidney injury within a large randomized trial, coronary artery bypass surgery done without a bypass pump reduced the relative risk of acute kidney injury by 17% (95% confidence interval, 3%-28%) compared with surgery done with a bypass pump, but without evidence of better preserved kidney function, mortality, or any other relevant clinical outcome 1 year later (with a baseline rate of 15%, a relative risk increase of 17% represents an absolute risk increase of about 2.5%).^19^

### Statistical Analysis

As recommended, we will conduct both per-protocol and intention-to-treat analyses^22,23^; however, the per-protocol analysis will be considered primary. We will report the stability of the noninferiority conclusion between the per-protocol and intention-to-treat results,^22,23^ and congruence in results between these analyses will increase confidence in our interpretation of the findings. Per-protocol analyses are often preferred for addressing questions of noninferiority because compared with intention-to-treat analyses they reduce the risk of falsely declaring noninferiority when a meaningful difference between the effects of 2 interventions in truth exists. In contrast, intention-to-treat analyses are preferred for questions of superiority because they reduce the risk of falsely declaring superiority when the effects of the interventions are, in truth, similar. As outlined in the TRICS-III main protocol, the per-protocol analyses will exclude (1) patients whose adherence to the assigned transfusion strategy is less than 90% and/or (2) patients who withdraw from the study at any time (including withdrawal of the patient by their treating physician). Currently, we anticipate an estimated 10% of randomized participants will be excluded from the per-protocol analysis; this subgroup of patients will be examined carefully to assess the potential for bias in the per-protocol analysis. In the intention-to-treat analyses (described below), all participants will be analyzed according to their original randomized group assignment regardless of whether transfusion occurred or which transfusion approach was followed. We will report the unadjusted absolute risk difference between the 2 groups with a 95% confidence interval using the large-sample normal approximation for a difference in proportions. We will accept noninferiority if the upper limit of the 2-sided 95% confidence interval for the difference in proportions of acute kidney injury (proportion of acute kidney injury in the restrictive-approach group minus the proportion in the liberal-approach group) is less than the noninferiority margin of 3.5%. We expect that data on postoperative serum creatinine will be missing for <2% of participants due to death and <5% due to missing data. For patients who were randomized but are missing at least 1 postoperative serum creatinine value, we will use recommended model-based multiple imputation methods using all available data to impute acute kidney injury status.^24-26^

#### Subgroup of preexisting chronic kidney disease

Compared with patients with preserved kidney function, patients with chronic kidney disease have a higher risk of acute kidney injury, as well as higher rates of anemia, perioperative mortality, and cardiovascular events.^27-32^ We will examine whether the presence of preexisting chronic kidney disease (primarily defined by an eGFR <60 mL/min/1.73 m2, with other eGFR cut-points examined in additional analysis) modifies the effect of a restrictive versus liberal approach to transfusion with respect to the risk of acute kidney injury.^30^ The *P* value for the interaction will be assessed by including the type of transfusion approach, an indicator variable for chronic kidney disease, and an interaction term (chronic kidney disease × type of transfusion approach) as independent variables in a regression model for binary outcome data. We will also present the absolute risk difference between intervention groups within each subgroup.

#### Other prespecified analyses

As outlined below, we will perform several supporting analyses including a complete-case analysis, an inverse probability weighted analysis, an intention-to-treat analysis, and an adjusted analysis, examining whether there is concordance with the primary analysis.^24-26^ We will also examine death as a competing outcome and examine alternative definitions of acute kidney injury.

#### Complete-case analysis

We will perform a complete-case analysis (no use of postoperative serum creatinine imputation) restricted to patients with at least 1 postoperative serum creatinine measurement (which will be over 90% of patients in the primary analysis). We may also perform an analysis restricted to those centers with over 90% of patients with complete serum creatinine measurements.

#### Intention-to-treat analysis

The intention-to-treat analysis will follow the same analytic approach as the primary analysis, but all participants will be analyzed according to their original randomized group assignment regardless of whether transfusion occurred or which transfusion approach was followed. In addition, if noninferiority is shown, an intention-to-treat superiority comparison will be performed using a 2-sided alpha of 0.05.

#### Adjusted analysis

In our experience with CORONARY and POISE kidney substudies,^19,26^ the unadjusted and adjusted results were virtually identical; nonetheless, we will conduct the following mixed-effects model for binary outcome data, considering center as a random effect (randomization stratum), adjusted for several prespecified factors (measured before randomization) based on their known association with acute kidney injury in the cardiac surgery setting: age (per year), sex, hemoglobin (per 10 g/L), left ventricular systolic ejection fraction categories, diabetes mellitus, treated hypertension, and eGFR (above or below 60 mL/min/1.73 m^2^).^33-35^ We will report the adjusted absolute risk difference with a 95% confidence interval (the latter estimated using bootstrap methods).^19,36^

#### Competing event of death

To determine whether the primary result is robust when the competing event of death is considered (which may influence serum creatinine measurements after surgery), we will also examine a composite outcome of acute kidney injury or death within 5 days of surgery.

#### Alternative definitions of acute kidney injury

We will repeat the primary analysis using alternative definitions of perioperative acute kidney injury including staging definitions,^20,37,38^ and assess the consistency of effect across stages of acute kidney injury based on visual inspection of point estimates and 95% confidence intervals. Despite the large sample size, these supplementary analyses for severe stages of acute kidney injury will have limited statistical power for small effects.

## Recognized Limitations

The primary outcome in this kidney substudy of TRICS-III is perioperative acute kidney injury (defined as an acute rise in serum creatinine concentration from preoperative values).^20^ While virtually all prevention trials of acute kidney injury follow this definition, this outcome is a surrogate endpoint that may not directly impact how a patient feels, functions, or survives. Detailed information on long-term permanent kidney function after hospital discharge will not be available in this trial. Nonetheless, our primary outcome follows the current recommended standard of diagnosis of acute kidney injury.^20^ We will also examine whether intervention effects are consistent across stages of acute kidney injury; despite being less frequent, events of Kidney Disease: Improving Global Outcomes (KDIGO) stage 2 and stage 3 acute kidney injury are more relevant to patients and health care providers. Although it is not possible to blind patients or providers to the intervention allocation, any resulting ascertainment bias should be minimal as we have the same prespecified serum creatinine measurement schedule for patients in both groups. Furthermore, our outcomes will be objectively measured by laboratory technicians unaware of group allocation.

## Conclusions

Acute kidney injury is a common and important complication of cardiac surgery. The TRICS-III kidney substudy will efficiently and reliably assess the renal effects of a restrictive versus liberal approach to red blood cell transfusion in the presence of acute anemia. It will also assess whether the effects of these approaches differ in patients with and without preexisting chronic kidney disease. Finally, this trial will provide some insights on the interplay between acute anemia, transfusions, and the risk of acute kidney injury.
